# Protective Performance of Polyaniline-Sulfosalicylic Acid/Epoxy Coating for 5083 Aluminum

**DOI:** 10.3390/ma11020292

**Published:** 2018-02-13

**Authors:** Suyun Liu, Li Liu, Fandi Meng, Ying Li, Fuhui Wang

**Affiliations:** 1 Corrosion and Protection Division, Shenyang National Laboratory for Materials Science, Institute of Metal Research, Chinese Academy of Sciences, Shenyang 110016, China; syliu13s@imr.ac.cn (S.L.); fdmeng@imr.ac.cn (F.M.); liying@imr.ac.cn (Y.L.); fhwang@imr.ac.cn (F.W.); 2 School of Materials Science and Engineering, University of Science and Technology of China, Hefei 230000, China; 3 Key Laboratory for Anisotropy and Texture of Materials (Ministry of Education), School of Material Science and Engineering, Northeastern University, Shenyang 110819, China

**Keywords:** polymer coatings, polyaniline, aluminum, EIS

## Abstract

Epoxy coatings incorporating different content of sulfosalicylic acid doped polyaniline (PANI-SSA) have been investigated for corrosion protection of 5083 aluminum alloy in 3.5% NaCl solution. The performance of the coatings is studied using a combination of electrochemical impedance spectroscopy (EIS), open circuit potential (OCP), gravimetric tests, adhesion tests, scanning electron microscopy (SEM) and X-ray photoelectron spectroscopy (XPS). The results demonstrate that the content of PANI-SSA not only affects the coating compactness and the transportation of aggressive medium, but also has a significant influence on the-based aluminum. The coating with 2 wt. % PANI-SSA exhibits the best corrosion inhibition due to its good protective properties and the formation of a complete PANI-SSA induced oxide layer.

## 1. Introduction

The 5083 aluminum alloy has a number of desirable features including low density, high strength and good welding performance, which is used in several components of ships. However, the aggressive ion Cl^−^ existed in marine environments can induce pitting corrosion on aluminum alloy [1,2,3,4]. Various surface treatment techniques have been used to enhance the corrosion resistance of aluminum alloys, such as chromate conversion coatings, anodic oxidation, and micro-arc oxidation [5,6,7]. However, these techniques are very complex, and the toxicity of chromates pollutes the environments. Developing new methods of pretreatment and primer for aluminum alloys is very important. 

Corrosion protection of polyaniline (PANI) coating for metals has been receiving increasing attention as potential components of corrosion-resistant coating system for years because of its specific properties, such as mechanical strength, electrical conductivity, corrosion stability, especially its oxidation-reduction reversibility [8,9,10,11,12,13,14]. There are different oxidation states for PANI, the leucoemeraldine base form (LB) corresponding to fully reduced state, the pernigraniline base form (PB), fully oxidized state and the emeraldine base form (EB). Simi-oxidized EB is the most stable state for polyaniline in atmosphere conditions. Depending on the PH of the solution, EB can be protonated in varying degrees by inorganic and organic acid and result in the formation of the emeraldine salt (ES) which may have a galvanic interaction with the metal substrate base [15,16,17,18,19].

A number of studies [15,20,21,22,23,24,25,26] on the corrosion inhibition properties of PANI coatings have focused on the protection of metals. PANI coating system has exhibited good corrosion resistance even after long term immersion in NaCl solution, especially after compounding with the epoxy resin. Y. Chen and the coworkers [27] investigated the anticorrosion performances of EB/epoxy coating on mild steel and found EB/epoxy coating can offer efficient corrosion protection after 40 days immersion in NaCl solution. Additionally, it is suggested that PANI improves the mechanical properties of epoxy resin. Jia et al. [25,26] studied the influence of dodecylbenesulfonic acid (DBSA) doped PANI on epoxy resin and found that the curing reaction and mechanical properties of epoxy were enhanced due to the interaction between epoxy and DBSA doped PANI. 

In fact, the inherent nature of the dopant anion associated with the PANI is critical in determining the level of corrosion protection afforded to metals [12,15,20,28,29,30,31,32,33]. Different acid-doped PANI has different protective properties. Results showed that organic acids doped PANI can provide a better protection because of its high conductivity and good redox reversibility. Generally, the anodic protection is believed to occur by galvanic interaction between ES and the metal substrate, leading to polarization of the metal to more positive potential along with the formation of EB and the subsequent release of the dopant acid anion. Sadegh Pour-Ali [34] stated that epoxy coating containing camphorsulfonate acid doped PANI exhibited good protective properties because of the formation of a PANI induced thickened oxide layer and iron-sulfonate complex at the coating/metal interface. Gupta [15] studied the corrosion protection of AA2024-T3 aluminum by Polyaniline-lignosulfonate/epoxy coating and found the protection performance was largely increased after the addition of Polyaniline-lignosulfonate. Corrosion inhibition was enhanced due to a thickened oxide layer and aided by the formation of the aluminum-sulfonate complex. Similar conclusions were also made by other researchers [20,25,35,36]: corrosion inhibition by organic acid doped PANI coatings was enhanced due to the formation of a PANI induced oxide layer. Dopants in ES also plays an important role in corrosion protection by forming a metal-dopant interface.

Among different kinds of organic acid doped PANI, sulfosalicylic acid doped polyaniline (PANI-SSA) is thought to be the most promising candidate of corrosion protection because of its specific properties. In general, PANI-SSA is widely used in the field of supercapacitors due to its super electrical conductivity. Gavrilov and coworkers [37] revealed that PANI-SSA could increase the specific capacitance of nanostructured carbon materials because of a remarkably higher conductivity. In addition, Janosevic et al. [38] also studied the use of PANI-SSA to carbonize the nanorobs/nanotubes and get a better electrocatalytic activity. More importantly, Morks [39] found sulfosalicylic acid is a suitable electrolyte for anodic film growth on aluminum surface. Therefore, it is reasonable to believe that PANI-SSA may give rise to the protective oxide film on the aluminum alloy base because of its good conductivity and redox reversibility. However, the application of PANI-SSA in the field of anti-corrosion for aluminum is not yet known. 

In this work, the protective properties of the epoxy coatings with different content of PANI-SSA have been compared. The fitting content of PANI-SSA in the epoxy coatings is ascertained. The effect of PANI-SSA concentration on the protective properties of the coatings and the oxide layer formed on the aluminum base was also discussed. 

## 2. Results

### 2.1. OCP Measurement

**Figure 1:**
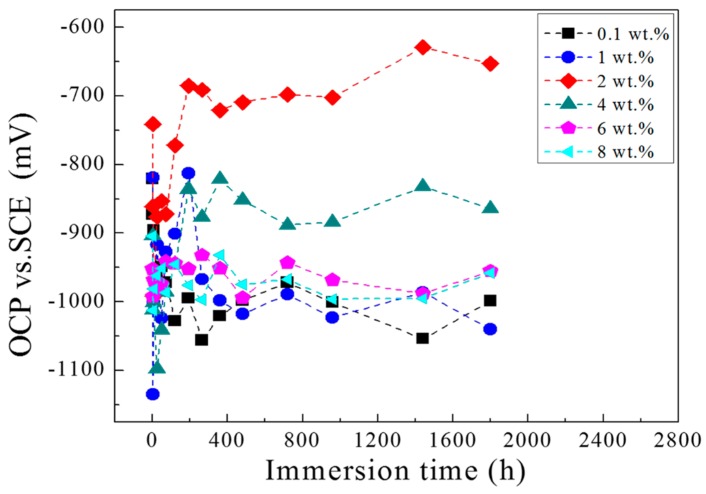
OCP (open circuit potential) curves for different concentration of PANI-SSA coated 5083 aluminum in 3.5% NaCl solution for 80 days.

OCP measurement cannot only provide direct information on the change of the surface morphology during immersion, but also indicates the protective properties of the coatings. Figure 1 shows the OCP evolutions of different PANI-SSA content of coatings/aluminum in 3.5% NaCl solution. For all the coatings/aluminum system, the OCP shifted negative quickly at first, 0.1, 6 and 8 wt. % coatings/aluminum kept slightly dropping in the whole measurement. While for the coatings with 1, 2 and 4 wt. % PANI-SSA, the OCP increased gradually after the initial immersion time. Especially for the coating with 2 wt. % PANI-SSA, its OCP decreased from −741 mV to −992 mV only after 6 h immersion, then increased gradually from −992 mV to −685 mV and stayed almost steady in the following measurement. This increase in OCP may be due to the formation of oxide film on the aluminum base beneath the coating with 2 wt. % PANI-SSA [40].

### 2.2. Electrochemical Impedance Spectroscopy (EIS)

**Figure 2:**
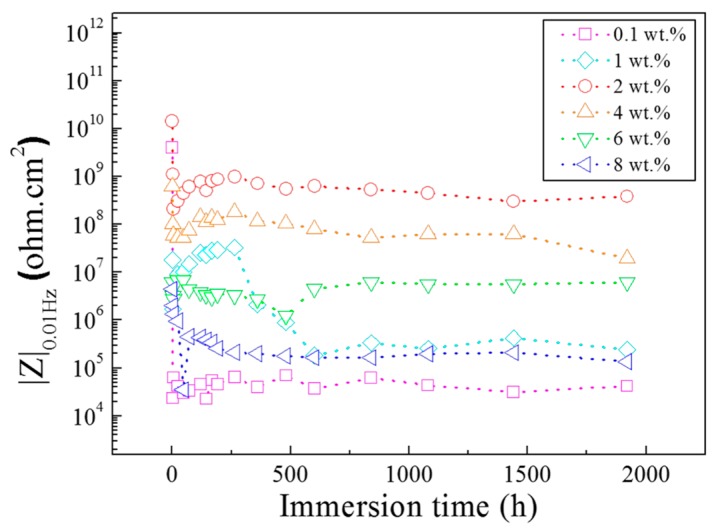
Evolution of |Z|_0.01Hz_ with immersion time in 3.5% NaCl solution for the coating/aluminum system.

To gain further insight into the corrosion protection of the coatings with different content of PANI-SSA, the EIS measurement was performed in 3.5% NaCl solution for 80 days. The magnitudes of the impedance modulus at 10 mHz were determined from the Bode magnitude data as a function of immersion time. The plot of |Z|_0.01Hz_ against immersion time for the coatings is shown in Figure 2. According to the Reference [27,35,41,42], the impedance modulus at low frequency (|Z|_0.01Hz_) is an appropriate parameter for characterization of the coatings corrosion protection. In present study, the |Z|_0.01Hz_ for the coatings with 0.1, 1, 2, 4, 6 and 8 wt. % PANI-SSA was 3.98 × 10^9^, 1.80 × 10^7^, 1.43 × 10^10^, 6.21 × 10^8^, 6.28 × 10^6^, 4.46 × 10^6^ ohm·cm^2^, respectively, after 1 h immersion. At initial immersion time, the |Z|_0.01Hz_ value mainly reflected the pore resistance of the coatings, which results from water and electrolyte penetration to the coating/metal interface [27,43,44]. Therefore, it is confirmed that the coating with 2 wt. % PANI-SSA has much better barrier property to corrosion ions than the other PANI-SSA content coatings. When the content of PANI-SSA was under 2 wt. %, the coating barrier property enhanced with the increase of PANI-SSA content in the coatings (Figure 2). While when PANI-SSA content increased further to 4, 6 and 8 wt. %, the |Z|_0.01Hz_ decreased along with the increase of PANI-SSA content. Besides, when immersion time increased to 6 h, the |Z|_0.01Hz_ of coatings with 0.1, 1, 6 and 8 wt. % PANI-SSA decreased rapidly to 6.28 × 10^4^, 3.51 × 10^6^, 2.74 × 10^6^ and 1.34 × 10^6^ ohm·cm^2^, respectively. The |Z|_0.01Hz_ of the coating with 2 wt. % PANI-SSA decreased rapidly in initial 6 h, and then increased gradually from 2.08 × 10^8^ ohm·cm^2^ at 1 day to 9.94 × 10^8^ ohm·cm^2^ at 12 days, and then stayed a plateau up to the final immersion time.

**Figure 3:**
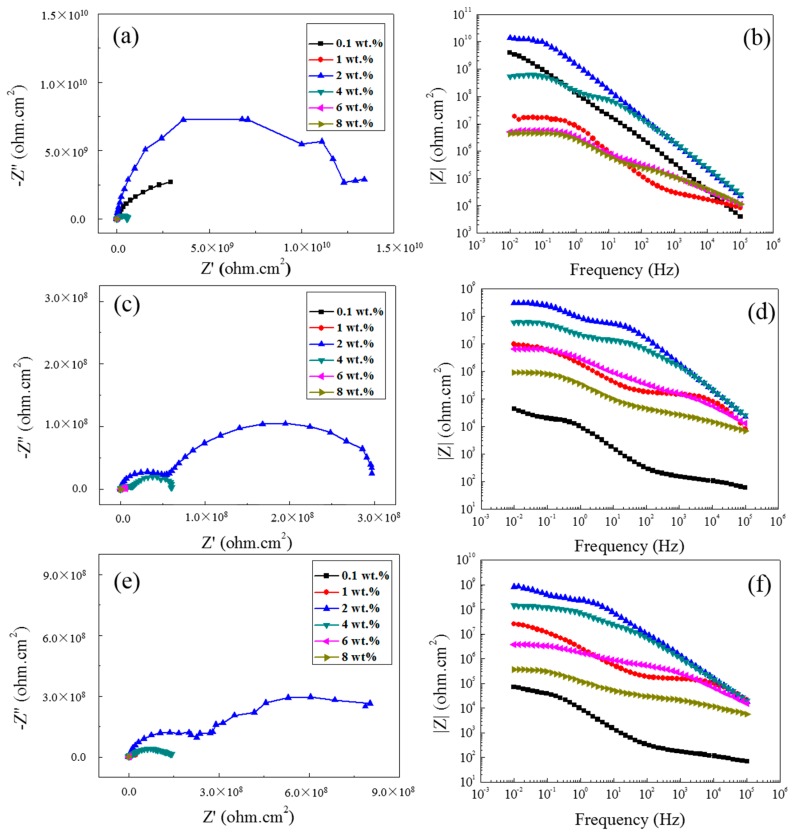
Nyquist plots of the coatings with different content of PANI-SSA after different immersion time in 3.5% NaCl solution: (**a**) 1 h; (**c**) 1 d; (**e**) 5 d and the corresponding Bode plots: (**b**) 1 h; (**d**) 1 d; (**f**) 5 d.

To explain the difference of the coatings with different content of PANI-SSA in the initial immersion time, the Nyquist plots and the corresponding bode plots after different immersion time in 3.5% NaCl solution are presented (Figure 3). The results show that the protective properties of different coatings change with immersion time. At the initial 1 h immersion time, there was only one capacitive loop for the coatings with 0.1 wt. % and 2 wt. % PANI-SSA, and the value of |Z|_0.01_ is higher which indicates a better barrier properties than other content of coatings. While the protective properties of the coatings with 0.1 wt. % PANI-SSA declined sharply after 1 day immersion. The coatings with 6 wt. % and 8 wt. % PANI-SSA also yield bad barrier properties and declined gradually with the immersion time increased. For the coatings with 2 wt. % and 4 wt. % PANI-SSA, there is an increase on the value of |Z|_0.01_. This may be due to the formation of the oxide film on the aluminum base beneath the coatings.

**Figure 4:**
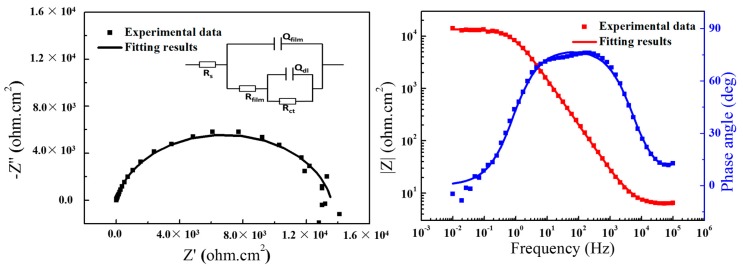
EIS (Electrochemical Impedance Spectroscopy) results of the uncoated aluminum in 3.5% NaCl solution.

Figure 4 shows the initial EIS results of the uncoated aluminum sample. As depicted in Figure 4, the Nyquist plots of uncoated aluminum revealed two capacitive loops, at high frequency the capacitive loop corresponding to the natural-formed passive film and the low frequency related to the charge transfer reaction, respectively. The simulating equivalent circuits used to fit the EIS data are also represented. Rs, Q_film_, R_film_, Q_dl_ and R_ct_ represent electrolyte resistance, capacitance of the film, resistance of the film, double layer capacitance and charge transfer resistance, respectively. The |Z|_0.01Hz_ of the uncoated aluminum sample at initial 1 h immersion time was 1.35 × 10^4^ ohm·cm^2^, smaller than the values of the coatings. Thus, the epoxy coatings with different content of PANI-SSA can protect 5083 aluminum alloy from corrosion.

### 2.3. Surface Morphology of the Coatings

**Figure 5:**
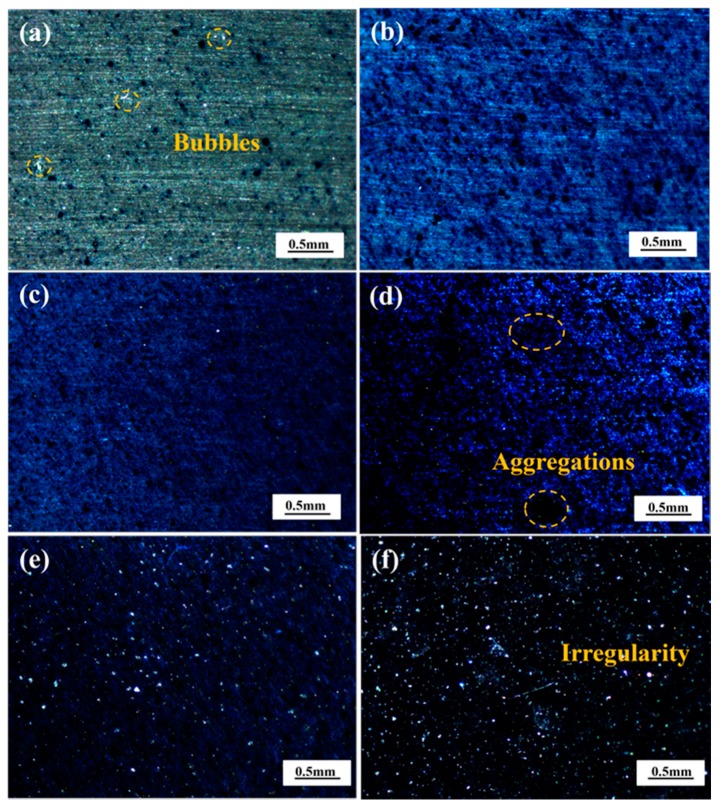
The stereo imaging characteristics of coatings with different content of PANI-SSA: (**a**) 0.1 wt. %; (**b**) 1 wt. %; (**c**) 2 wt. %; (**d**) 4 wt. %; (**e**) 6 wt. %; (**f**) 8 wt. %.

The stereo imaging characteristics of the epoxy coatings with different content of PANI-SSA are seen in Figure 5. Some small bubbles were observed on the surface of coating with 0.1 wt. % PANI-SSA from Figure 5a. The bubbles continuously disappeared as the concentration of PANI-SSA increased. It can be seen the coatings with 1 wt. % (Figure 5b), 2 wt. % (Figure 5c) and 4 wt. % (Figure 5d) PANI-SSA exhibited smooth surface with homogeneous dispersion of the PANI-SSA fillers. While when the content was above 4 wt. %, serious aggregations of PANI-SSA were formed. Uneven surfaces were seen from the stereo imaging characteristics of the coatings with 6 wt. % (Figure 5e), 8 wt. % (Figure 5f) PANI-SSA, which was caused by the poor dispersion and serious aggregations of PANI-SSA fillers. The coatings consist of only three components: epoxy resin and PANI-SSA fillers, the epoxy resin is uniform and parent, so the dark patch seen in the coatings should be the PANI-SSA aggregations. These bubbles and aggregations may accelerate the failure of the coatings.

### 2.4. Gravimetric Results

**Figure 6:**
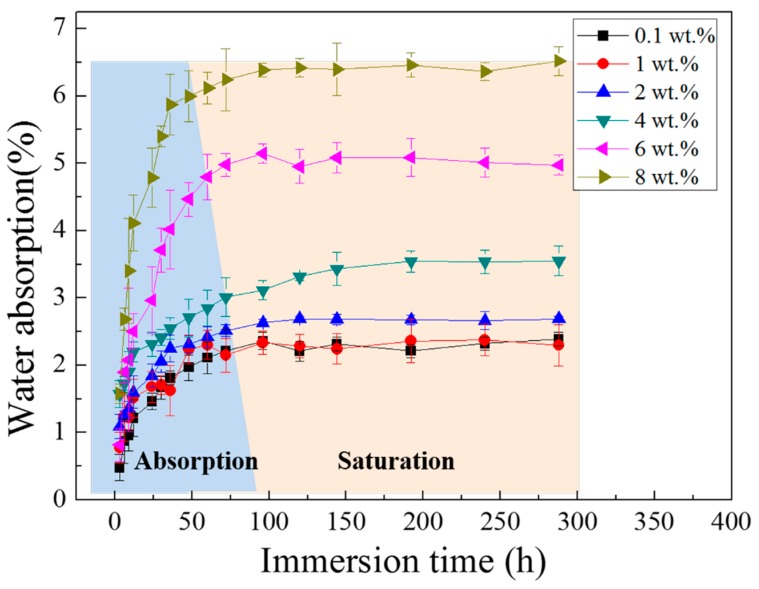
Water absorption curves for the free film samples in 3.5% NaCl solution for different immersion period.

The study of water absorption is of great importance, since the penetration of water is the main reason for coating’s degradation [45]. By gravimetric tests, the mass of absorbed water in the different content PANI-SSA coating can be calculated. The water absorption (*Q_t_*) curves in Figure 6 show the weight gain of the coatings after different immersion period. The *Q_t_* of 0.1, 1, 2 and 4 wt. % of PANI-SSA coatings showed a rapid increase in mass within the initial 48 h immersion, after which a gradual growth was observed in 144 h, and finally remained steady till the end of the experiment. While for the coatings with 6 wt. %, 8 wt. % PANI-SSA, the trend of *Q_t_* included a rapid increase within 60 h, and then remained steady until the end of the experiment. From the results, it is clear that water diffusion was accelerated by the addition of PANI-SSA to the epoxy coatings. Additionally, *Q_t_* increased with the PANI-SSA content increased. 

### 2.5. Adhesion Measurements

**Figure 7:**
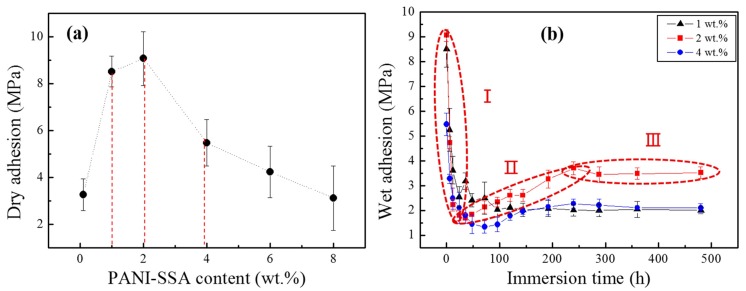
Adhesion results (**a**) with the increase of PANI-SSA content; (**b**) with immersion time for the coating/aluminum samples.

Since the adhesion is a basic requirement for good mechanical and physical chemistry properties in the coated metal surface [46,47,48,49]. The results of the dry adhesion measurements for the coating with different content of PANI-SSA are shown in Figure 7a. It can be seen that the 1, 2 and 4 wt. % of PANI-SSA coating showed better adhesion strength and the values were 8.51, 9.07 and 5.47 MPa, respectively.

Wet adhesion is an important parameter and significantly influences the properties of coating. The dry adhesion changes to be wet adhesion once was immersed in the electrolyte. Generally, adhesion measurements are carried out before and after different time of immersion periods and the wet adhesion decreases with the increase of immersion time. The wet adhesion of the 1, 2 and 4 wt. % coating/aluminum samples with the immersion time is shown in Figure 7b. In the present study, 2 wt. % PANI-SSA coating maintained relatively high wet adhesion strengths (3.52 MPa) after 480 h immersion, while for 1 wt. % and 4 wt. % PANI-SSA coating was 2.08 MPa and 2.10 MPa, respectively. It can be seen that the wet adhesion of 2 wt. % PANI-SSA coating performed three stages: the values dropped rapidly at the initial 50 h, increased during the 50 h to 250 h immersion time and stayed steady at the following immersion time. The increase of wet adhesion may be due to the enhanced bonding force between PANI-SSA coatings and the-based aluminum.

### 2.6. Tensile Results

**Figure 8:**
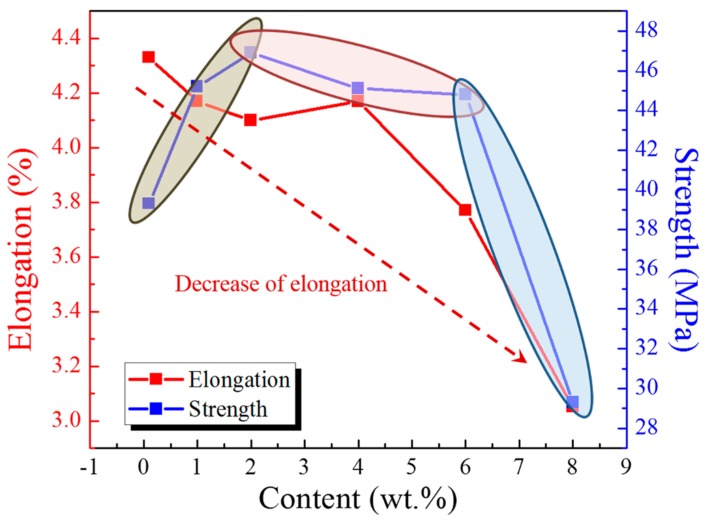
Tensile tests results (coating strength and elongation) of the coating with different content PANI-SSA.

The mechanical properties of the free film samples, including tensile strength and coating elongation, were investigated by tensile tests. The effect of PANI-SSA content on tensile strength and coating elongation has been evaluated and shown in Figure 8. It is observed that the maximum tensile strength is 46.9 MPa which achieved at the content of 2 wt. %, then decreased gradually to 44.8 MPa at 6 wt. % PANI-SSA content. When the content of PANI-SSA got to 8 wt. %, tensile strength fell to 29.3 MPa rapidly. The initial (when the amount of PANI-SSA under 2 wt. %) improvement of mechanical properties is correlated with the fracture surface features of the fillers, which is attributed to good interaction between epoxy and the small amount addition of PANI-SSA fillers. This interaction produces better interface bond between PANI-SSA and the epoxy matrix. In addition, the micro crack bridging through PANI-SSA and resin toughening enhances the strength of resin [50,51]. When the content of PANI-SSA in the coating was above 4 wt. %, the tensile strength of coating decreased with the increase of PANI-SSA content, which was mainly due to the aggregations of PANI-SSA fillers. The aggregations (Figure 5) in the coatings results in the interface de-bonding, fiber pull out, resin cracking, resin deformation and fiber breakage during the tensile tests. Consequently, tensile strength declines. Low fillers content also causes dramatic drop in the fracture strain, and the stain usually declines with the increasing fillers content [51]. In the present study, similar fracture stain decrease was seen as long as the fillers content increased. The results were observed in Figure 8, which shows the changes of coating elongation with the increase of PANI-SSA fillers. The degree of elongation dropped from 4.33% to 3.05% when the content of PANI-SSA increased from 0.1 wt. % to 8 wt. %. This deformation of the coating is mainly due to the rigid nature of PANI-SSA fillers and its aggregations in the epoxy resin [51,52].

### 2.7. Surface Analysis of the Based Aluminum

**Figure 9:**
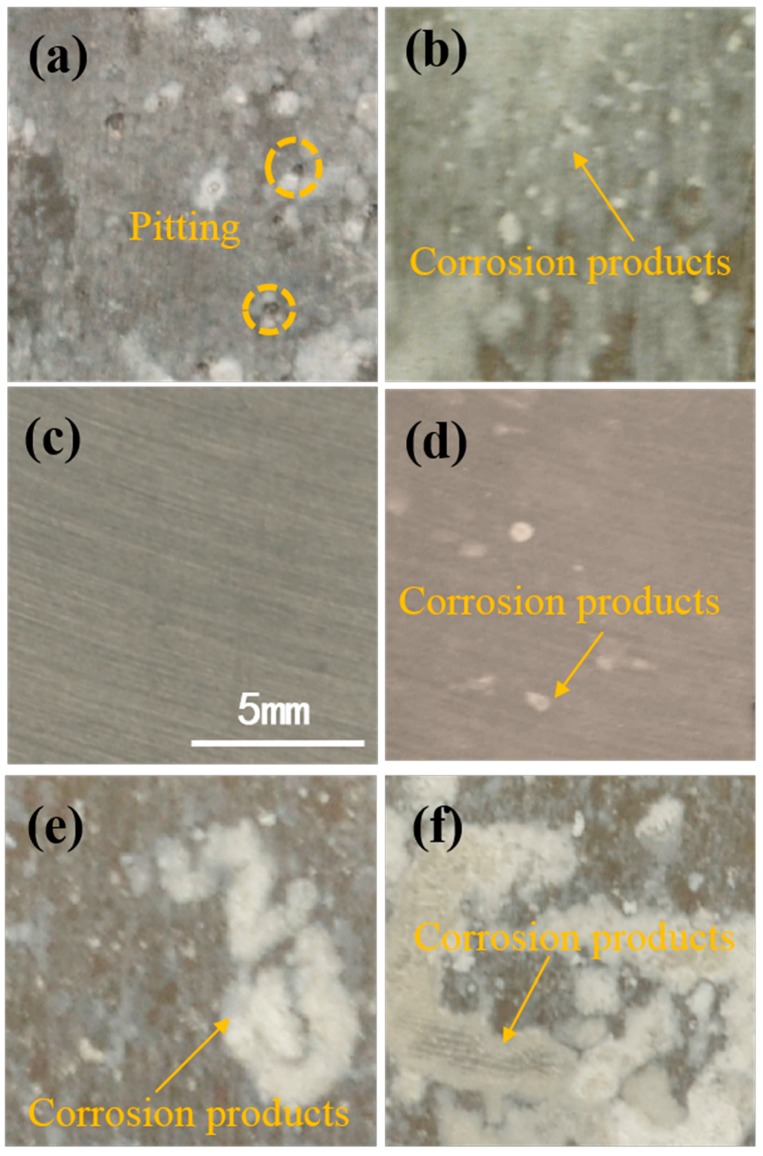
Macro images of corrosion morphology of the 5083 aluminum based alloy beneath coatings with different content of PANI-SSA (**a**) 0.1 wt. %; (**b**) 1 wt. %; (**c**) 2 wt. %; (**d**) 4 wt. %; (**e**) 6 wt. %; (**f**) 8 wt. % after immersion.

**Figure 10:**
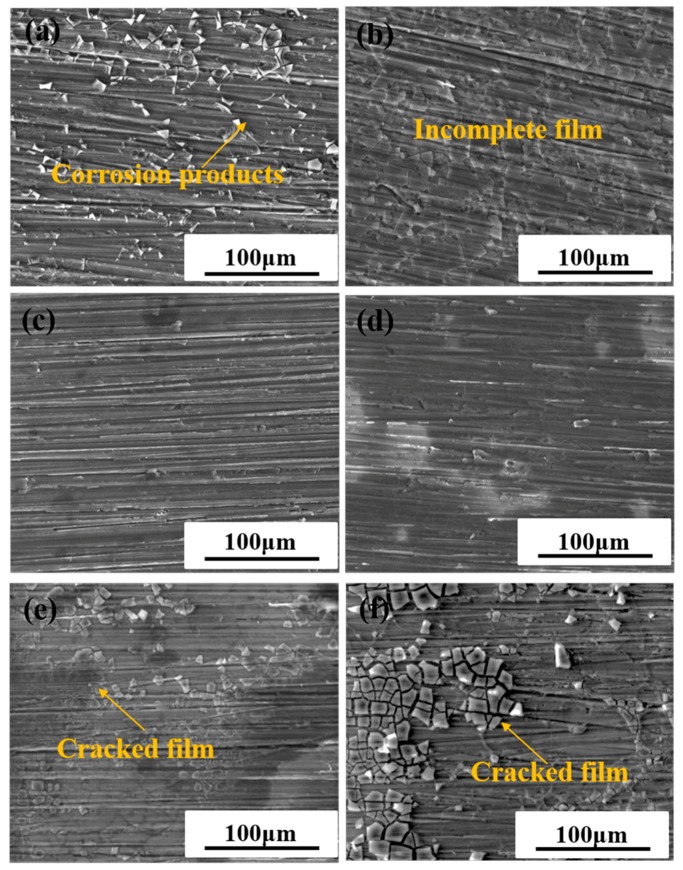
SEM images of corrosion morphology of 5083 aluminum alloy beneath coatings with different content of PANI-SSA (**a**) 0.1 wt. %; (**b**) 1 wt. %; (**c**) 2 wt. %; (**d**) 4 wt. %; (**e**) 6 wt. %; (**f**) 8 wt. % after immersion.

In order to further understand how the coatings and the content of PANI-SSA fillers affects the aluminum surface base, macroscopic and microscopic morphologies of the aluminum substrate beneath the coatings with different content of PANI-SSA after 80 days immersion were investigated. Figure 9 and Figure 10 shows the optical images and SEM images of the surfaces of aluminum base after 80 days immersion in 3.5% NaCl solution. All the coatings were peeled off before the surface analysis.

The corrosion morphologies of the 5083 aluminum-based alloy beneath PANI-SSA coatings are shown in Figure 9. Many corrosion products were seen beneath the coatings with 0.1, 1, 6 and 8 wt. % PANI-SSA, while no severe corrosion except a few localized corrosion pitting was observed on the aluminum base when the PANI-SSA content is 4 wt. %. The surface beneath the coating with 2 wt. % PANI-SSA was still quite glossy with no visible corrosion pits.

**Figure 11:**
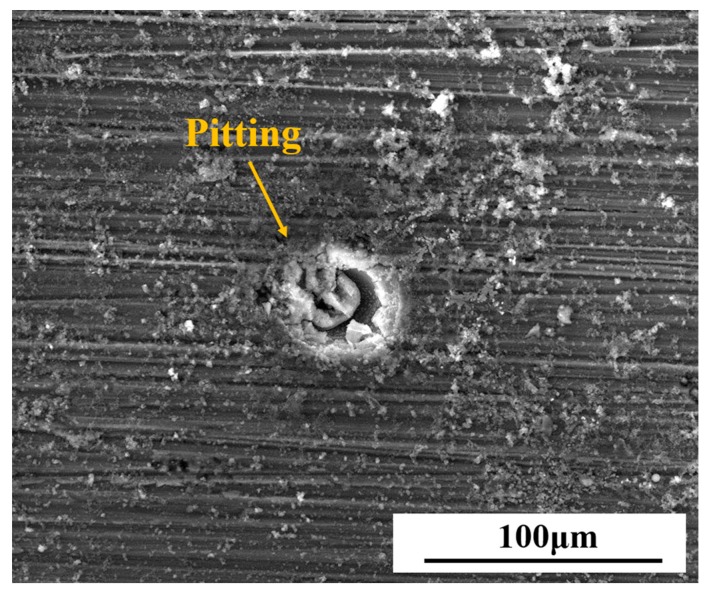
SEM images of corrosion morphology of uncoated 5083 aluminum alloy after 30 days immersion in 3.5% NaCl solution.

Figure 10 shows the microscopic morphologies of the aluminum base beneath different content of PANI-SSA coatings. As a contrast, the uncoated aluminum sample was immersed in 3.5% NaCl solution for 30 days, the microscopic morphologies of the metal surface was shown in Figure 11. Pitting corrosion along with the appearance of the flocculent corrosion products can be observed. Serious pitting occurred on the 5083 aluminum alloy when there is no coating. While the 2 wt. % PANI-SSA coated surface was rough with little polishing grooves. Some intact corrosion products film along the polishing grooves was also observed. However, the corrosion products layer beneath the coating with 0.1 wt. % and 1 wt. % PANI-SSA were not integral. Besides, some cracks along with the product layer was formed on the aluminum base when PANI-SSA content is 6 wt. % and 8 wt. %. With the PANI-SSA content increases, products layer becomes thicker along with more cracks. Obviously, some corrosion pits were even seen on the surface aluminum beneath the coatings with 0.1, 1 and 8 wt. % PANI-SSA.

**Figure 12:**
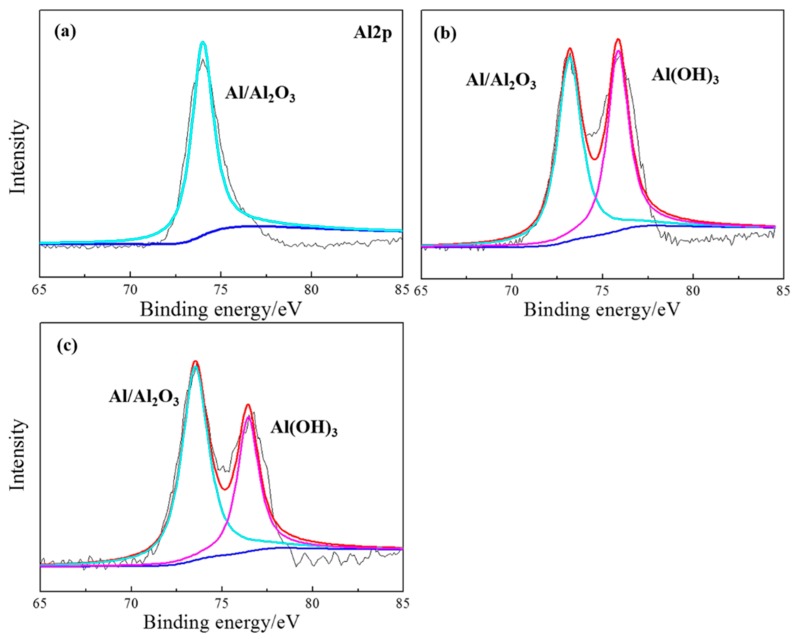
XPS results of the aluminum base beneath coatings with different content of PANI-SSA (**a**) 1 wt. %; (**b**) 2 wt. %; (**c**) 4 wt. % after electrochemical impedance measurement.

The base surface beneath the coating with 1, 2 and 4 wt. % PANI-SSA was analyzed using XPS analysis following the electrochemical impedance measurements (in 3.5% NaCl solution). The main component of the coated surface is Al and O. The results of the photo-electron signals after background subtraction and the curve fitting are shown in Figure 12. It shows the Al2p spectra of the 5083 aluminum-based alloy surface. There are only one peaks which are Al/Al_2_O_3_ at 73.5 eV for the aluminum base beneath the coating with 1 wt. % PANI-SSA, while two peaks which are Al/Al_2_O_3_ at 73.5 eV and Al(OH)_3_ at 76.4 eV beneath the coatings with 2 wt. % and 4 wt. % PANI-SSA. With the increase of PANI-SSA content, the coatings became less porous (shown in Figure 5c), this relatively tight structure retarded the transportation of aggressive medium like Cl^−^, hence promote the formation of PANI oxide layer. Ref. [8,9,15] also stated that PANI can act as a catalyst for oxidation of metal, leading to the formation of a passive, protective metal hydroxide layer. When the content of PANI-SSA was over 2 wt. % (up to 4, 6 and 8 wt. %) the PANI-SSA became more agglomerated which may build a ‘express entry’ for Cl^−^ to get to the coating/aluminum interface and destory the coating adhesion. Therefore, preventing the contact between the aluminum substrate and the PANI-SSA fillers, and retarding the redox reversibility of PANI-SSA, resulting in less oxidation reaction. There is much more oxide product with the increase of PANI-SSA content. Consequently, it can be concluded that PANI-SSA accelerate the oxidation of aluminum alloy.

## 3. Discussion

After studying the electrochemical behavior of the coatings with different content of PANI-SSA on 5083 aluminum alloy for 80 days immersion time in 3.5% NaCl solution, it is found that the content of PANI-SSA not only affects the original coating surface, mechanical properties and protective performance of the coatings, but also has a significant influence on the surface morphology of the aluminum base. The optimum content of PANI-SSA in epoxy coating is 2 wt. %. The underlying mechanisms are discussed as follows.

### 3.1. Influence of PANI-SSA Content on Protection Properties of Coating

The impedance results (Figure 2) indicated that the coatings with low and high concentration of PANI-SSA show a poor coating resistance. Whereas at the optimum concentration (2 wt. %) the coating shows the best protective performance, which is different from the results revealed by Gupta (the optimum concentration is 5 wt. %) [15] and Chen (the fitting content is 10 wt. %) [27]. Different factors can account for this consequence. Microscopic morphology observation of the coatings samples revealed the presence of porosity (Figure 5a,b) in lower content of PANI-SSA (0.1 and 1 wt. %). While PANI-SSA fillers begin to aggregate (Figure 5d–f) when the content of PANI-SSA surpass 4 wt. %. The existence of bubbles in the coating (Figure 5a,b) makes it easier for aggressive particles to penetrate into the coating and reach the aluminum/coating interface. Meanwhile, the presence of water normally decreases adhesion between the coating and the substrate [53]. With the spread of water at the interface, blisters and under-film corrosion sites occur considerably [45,52,53]. In the present study, a rapidly adhesion decrease of the coating with 1 wt. % PANI-SSA can be seen and some pit corrosion occurs on the aluminum base. However, the aggregations of high content (4, 6 and 8 wt. %) of PANI-SSA fillers in the coating would be implicated in the water absorption as they appear loosely embedded in the resin. Hence, additional amount of water can be accommodated at the interface between these aggregations and the resin. This weak link between fillers and binders could also provide paths of facile diffusion towards to the aggregations. Then the micro-voids are quite probable to form due to a lack of impregnation of PANI-SSA with epoxy resin [42,50,54]. The weakly bonded interface caused by pores and agglomerations also affects the tensile strength and coating elongation of the coatings. This bad mechanical property in return accelerates the water absorption of coatings and results in the water gathering in the coating/metal interface, obviously aggravates aluminum corrosion. In the end, poor protective properties of inappropriate addition of PANI-SSA accelerates the failure of the epoxy coating. 

### 3.2. Influence of PANI-SSA Content on the Aluminum Surface Beneath Coating

The addition of PANI-SSA in the coating also affects the formation of PANI-induced oxide layer on the aluminum-based alloy. The SEM results showed in Figure 10 indicates the formation of a dense compact oxide film beneath the coating with 2 wt. % PANI-SSA, while little corrosion products but corrosion pit was found beneath the coating with 0.1 wt. % and 1 wt. % PANI-SSA. It is obvious the pores in the coating make it easy for Cl^−^ to diffuse into the coating and cause pitting on the surface base. Moreover, little addition of PANI-SSA may not provide enough reduction reaction to accelerate the oxidation of aluminum [15]. Indeed, Significant passivation of aluminum occurred when PANI-SSA content was above 4 wt. % (a thicker oxide layer appeared on the aluminum base shown in Figure 8). Meanwhile, a lot of cracks were also seen, the protective performance of the coating decreased remarkably (Figure 2) which corresponded to the decreased coating barrier properties. This was caused by the aggregations of PANI-SSA fillers in the coating (Figure 5). The aggregations of PANI-SSA also result in an increasing *Q_t_* (Figure 6) of coatings. Large amount of water absorption in the coating accelerates the transportation of Cl^−^ from the solution into the coating and to the coating/aluminum interface, and then destroys the adhesion between coating and substrate, separates the PANI-SSA coating from the aluminum substrate, finally causes severe corrosion on the aluminum-based surface.

The above SEM results, XPS results in combination with the OCP results indicates that a mixed Al/Al_2_O_3_/Al(OH)_3_ dense stable passive film is gradually formed on the aluminum base beneath 2 wt. % PANI-SSA coating. With the PANI-SSA content increases, the oxide film layer becomes thicker which can be confirmed from the SEM images (Figure 10). The passivation ability of PANI can also be evidenced from the shift of OCP in noble direction (Figure 1). Many authors [8,15,27,55,56] had confirmed this anodic effect of PANI to metals. Under immersion conditions, oxidation of metal can act as a trigger for the reduction of the PANI and lead to the formation of a passive, protective metal oxide layer. The results was also supported by Fahlman et al. [57], they confirmed that PANI coating has the protection effect on metals by withdrawing the charge transfer of the metals and passivating its surface. The oxide film on the aluminum base in present study become thicker with the increase of the PANI-SSA content. However, excess addition of PANI-SSA fillers lowers the compactness of coatings that much more aggressive medium gets into the coatings. The aggressive medium spreads along the coating/aluminum interface and decreases the adhesion. Therefore, the destructive effect of Cl^−^ to the aluminum oxide dominated that the aluminum surface was severely destroyed. As a result, it is found to be inhomogeneous, consisting of a number of pitting underneath the high content (8 wt. %) PANI-SSA coating. Furthermore, the oxide layer on the aluminum base became cracked when the PANI-SSA in the coatings increased. Accordingly, Cl^−^ would destroy the passive film on aluminum alloy and form pitting due to dominating Cl^−^ attack. Z. Szklarska-Smialowska [2] also discussed the aluminum passive films are weakened in chloride solution. In this work, the relatively complete PANI induced oxide layer was formed on the aluminum base beneath the coating with 2 wt. % PANI-SSA. This is mainly because the barrier property of the coating is good, which weaken the destructive effect of Cl^−^.

### 3.3. The Fitting Content of PANI-SSA in Epoxy Coating

Generally, a uniform distribution of particles decreases the permeability of aggressive medium and increases the corrosion protective performance of coatings. With a uniform, compact structure and the resulting relatively lower water absorption, good adhesion and tensile strength, the epoxy coating with 2 wt. % PANI-SSA yields the highest protective properties. The epoxy coating with 2 wt. % PANI-SSA has the best corrosion protection because of its barrier properties and the PANI induced protective oxide layer on the aluminum base. S. Sathiyanarayanan [22] proposed that the positive OCP value was increased due to the redox interaction between the metal and the PANI film. In this work, The OCP of the coating with 2 wt. % PANI-SSA shifted to positive value after 1 day immersion. It means that the oxide film on the metals began to form once the solution reached the metal/coating surface [15,40,56]. Furthermore, only a uniform distribution and sufficient concentration of PANI-SSA fillers ensures the compactness of coatings and releases enough sulfonate anions (dopant) to assist the formation of passive layer [34,58]. The protection efficiency of oxide film beneath the coatings with excess content of PANI-SSA decreased mainly due to the presence of cracks and defects. From the empirical tests herein, a 2 wt. % PANI-SSA coating yields the highest barrier performance in 3.5% NaCl solution after 80 days immersion. 

**Figure 13:**
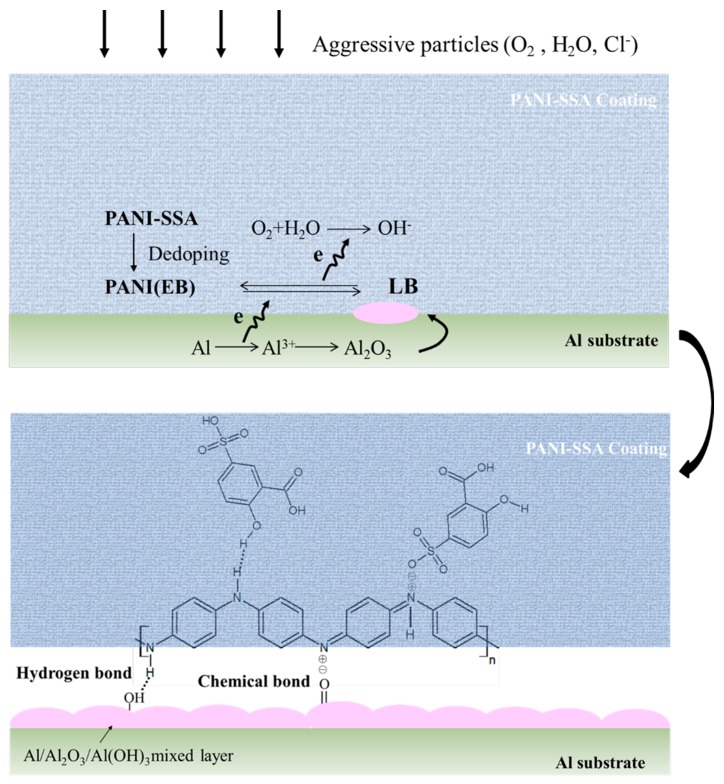
Schematic protection mechanism of the epoxy with PANI-SSA for 5083 aluminum alloy in 3.5% NaCl solution.

The schematic diagram demonstrated the growth of PANI-SSA induced oxide layer, which is shown in Figure 13. When aggressive particles (O_2_, H_2_O, Cl^−^) permeate into the PANI-SSA coating, PANI-SSA de-dopes to PANI (EB). The cyclic redox interaction occurs between EB and LB. In this reaction, EB serves as cathode along with the anodic dissolution of aluminum, thus EB is reduced to the LB. Then LB is re-oxidized to EB because of the oxidization effect of O_2_. Consequently, a protective oxide layer was formed on the coating/aluminum interface. As a result, this newly-formed hydrogen bonds, chemical bonds, barrier properties and the dense continuous oxide film enhance the protective properties of the epoxy coating with 2 wt. % PANI-SSA.

## 4. Materials and Methods

### 4.1. Sample Preparation

The substrate was 5083 aluminum alloy commonly used in marine environment, and the nominal chemical composition is shown in Table 1. The chemical composition of 5083 aluminum has been checked with energy-dispersive X-ray spectroscopy (EDS). The dimensions of the 5083 aluminum alloy specimens were 10 × 10 × 5 mm^3^ for EIS tests and 30 × 30 × 5 mm^3^ for adhesion tests. All aluminum alloy specimens were mounted in curing epoxy resin with an exposure area of 1 cm^2^ for EIS tests and 9 cm^2^ for adhesion tests. All the specimens were ground to 800 grit size using SiC paper, degreased with acetone and ethanol, and then put into desiccators for future use.

The E44 was dissolved in xylene at a ratio of 10:5, different content (0.1, 1, 2, 4, 6 and 8 wt. %) of PANI-SSA (diameter, 7–10 μm, conductivity, 2 s/cm, Taizhou yongjia trading company) was added to epoxy xylene solution, then stirring and ultrasounding for 3 h respectively to formulate coating system. Polyamide was chosen as the curing agent, its content was 80% weight of epoxy resin. The main component of the coating is C, H, O, N.

Two kinds of samples were prepared for the measurements: (1) the coating/aluminum sample, in which the coating was applied on the aluminum using a hand brush, and then cured in an oven under the following conditions: 40 °C for 4 h, 60 °C for 20 h, and room temperature (25 °C, 30% RH) for 168 h (7 days); (2) the free film sample, in which the coating was brushed on a silica gel plate, after being cured in an oven with 40 °C for 4 h, the film was peeled off from the plate and cut into specific dimensions for gravimetric and tensile tests, respectively. One of the dimension was 10 × 50 × 0.2 mm which was prepared for gravimetric experiment, another was cut into a special dimension which was used for tensile test, and the details of the dimension can be found in Reference [45]. Then the film continued to cure at 60 °C for 20 h and at room temperature (25 °C, 30% RH) for 168 h (7 days). The average dry coating thickness was found to be 100 ± 10 μm using a hand-held electronic gauge (PosiTector 6000 of Defelsko, Ogdensburg, NY, USA) according to ISO 2808-1997 [59]. Prior all tests, the samples were stored in desiccators to keep dry and avoid any changes in properties due to adsorption of moisture from the atmosphere.

### 4.2. EIS Measurements

To gain further insight into the corrosion protection afforded by the coatings with different content for aluminum and corrosion behavior of uncoated aluminum, an Autolab electrochemical station (Metrohm) was used for EIS measurements in 3.5% NaCl solution. A three-electrode cell was used. A platinum plate of size (20 × 20 mm^2^) was used as the counter electrode. The saturated calomel electrode (SCE) was selected as the reference electrode. A sinusoidal ac perturbation of 5 mV (rms) amplitude was used for the uncoated sample. According to the highly resistance of the coatings system at initial immersion time, the amplitude was 20 mV (rms). After 6 h immersion, the system became gradually stable; the amplitude was 10 mV (rms). The perturbation was coupled with the open circuit potential (OCP) and the EIS tests were performed over a frequency range of 100 kHz to 10 mHz. In order to minimize the ambient noise, all experiments were carried out in a faraday cage. Considering the stability of the measured systems, the device was kept for 5 min after moving the sample into the electrolytic cell. 

### 4.3. Adhesion Tests

The adhesion tests of epoxy coatings with different content of PANI-SSA for aluminum were conducted by PosiTest Pull-Off Adhesion Tester according to ASTM D4541-02 [60] (a standard about the evaluation method of the intensity which separated the coating from the metal surface). The samples were mounted in curing epoxy resin with an exposure area of 9 cm^2^ before applying coating on it to prevent any possible influence on the test area. The wet adhesion tests were carried out at different immersion periods. At least six parallel samples were conducted, and the average values were taken as the final result. The test dolly diameter was ϕ 20 mm.

### 4.4. Gravimetric Tests

Gravimetric data were obtained by measuring the mass of different free film samples at stipulated time intervals in 3.5% NaCl solution, respectively. After removal from the test solution, the surface was cleaned with the aid of filter paper and re-weighed. The maximum time out of the test solution was 30 s. The gravimetric tests were carried out using a Sartorius BS124S microbalance (1 μg resolution). The experiments were carried out five times to ensure reproducibility of the results. The water absorption (*Q_t_*) was then calculated according to the following equation [61]:

*Q_t_* = (*m_t_* − *m*_0_) × 100%/*m*_0_ (1)

where *m_t_* is the mass of free film at time *t*; *m*_0_ is the initial mass before immersion and *Q_t_* is the water absorption (%) at time *t*.

### 4.5. Tensile Test

To evaluate the properties of strength and the toughness of the coating with different content of PANI-SSA, the tensile was studied by universal tensile testing machine (AK20KN of Shimadzu, Kyoto, Japan) with a strain rate of 2 mm/min, according to ISO 37-2005 [62]. Six specimens were applied for every test and the mean value was chosen as the final result.

### 4.6. Analysis of Morphology of the Coatings and the Structure of Based Aluminum

The stereo imaging characteristics of coated substrates were analyzed via a stereoscopic microscope (ZEISS, Stemi 508, Zeiss, Upper Cohen, Germany).

The cross-section morphology of the coatings were characterized by scanning electron microscopy (SEM, Inspect F, Inspect F50, FEI Co., Hillsboro, OR, USA). After 80 days immersion and the final EIS test, the coatings on the aluminum were removed, the macroscopic and microscopic morphologies of substrate were imaged using a digital camera (Nikon, Tokyo, Japan) and SEM, respectively.

The different aluminum after 80 days immersion were characterized by X-ray photoelectron spectroscopy (XPS). XPS measurements were performed on Escalab 250 (Thermo Fisher VG, Shanghai, China). The base pressure was 2.4 × 10^−8^ Pa. Monochromatized Al Kα 1486.6 eV was used as the X-ray source. The calibration of the binding energy of the spectra was performed with the C1s (284.6 eV) peak. The Al2p spectra were further resolved into component peak by XPSPEAK software (Version 4.1, CUHK, Hong Kong, China).

## 5. Conclusions

The epoxy coatings with different content of PANI-SSA were successfully applied on 5083 aluminum alloy. The corrosion resistance, adhesion, water absorption, tensile strength and morphology were analyzed during the immersion periods in 3.5% NaCl solution for 80 days. At a low content of PANI-SSA, the coatings show poor compactness with little pores. Conversely, a high content and high volume fraction of particles degrade the coating protective performance owing to aggregations. Besides, a small addition of PANI-SSA would not have an obvious impact on water absorption and tensile strength of coatings. Only a uniform distribution and sufficient concentration of PANI-SSA fillers can ensure the compactness of coating and assist the formation of the passive layer on the aluminum. The coating with 2 wt. % PANI-SSA yields the highest values of coating pore resistance, and the coated surface presented a more compact microstructure. Corrosion inhibition of 2% PANI-SSA coating was enhanced due to its good protection properties and aided by the formation of a thickened oxide layer induced by PANI-SSA. 

## Figures and Tables

**Table 1:** Chemical composition (wt. %) of 5083 aluminum alloy.

Element	Si	Fe	Cu	Mn	Mg	Cr	Ni	Zn	Al
Content (wt. %)	0.12	0.25	0.1	0.55	4.46	0.07	0.05	0.05	bal
